# Global trends in scientific production on physical exercise and spinal cord injury

**DOI:** 10.3389/fspor.2025.1678162

**Published:** 2026-01-22

**Authors:** Gabriel de Souza Zanini, David Michel de Oliveira, Pedro Luiz Santorsula de Paula Oliveira, Danilo Alexandre Massini, Dalton Müller Pessôa Filho

**Affiliations:** 1 Department of Physical Education, School of Sciences (FC), São Paulo State University (UNESP), Bauru, Brazil; 2 Department of Physical Education, Faculdades Integradas de Jau (FIJ), Jau, Brazil; 3 Department of Physical Education, Federal University of Jataí (UFJ), Jataí, Brazil; 4 Postgraduate Program in Animal Bioscience (PPGBA), Institute of Health Sciences (ICS), Federal University of Jataí (UFJ), Jataí, Brazil; 5 Postgraduate Program in Human Development and Technology, São Paulo State University (UNESP), Rio Claro, Brazil

**Keywords:** adaptive physical activity, knowledge mapping, motor performance, research trends, wheelchair

## Abstract

**Background:**

Physical exercise (PE) plays a critical role in the rehabilitation of individuals with spinal cord injury (SCI), yet global scientific production in this field shows heterogeneous distribution across themes and collaboration networks. Scientometric analyses can clarify research evolution, collaborative structures, and thematic priorities. This study aimed to provide a comprehensive mapping of two decades of scientific output on physical exercise and SCI.

**Methods:**

Original research articles published between 2004 and 2024 were retrieved from Web of Science, PubMed, and Scopus using a standardized search strategy. Reviews, meta-analyses, editorials, letters, and conference abstracts, and gray literature, as well as out-of-scope studies were excluded. Data were analyzed with Bibliometrix (v4.1.3), VOSviewer (v1.0.0), and Microsoft Excel®.

**Results:**

A total of 692 original articles were included. The field exhibited a modest average annual growth rate (1.31%), with a publication peak in 2021 (*n* = 59), followed by a decline from 2022 onward. Keyword co-occurrence and conceptual structure analyses identified five dominant thematic axes: (i) physical and functional performance, (ii) physiological responses, (iii) clinical rehabilitation, (iv) assistive engineering and biomechanics, and (v) metabolism and health. Psychosocial dimensions and assistive technology–focused research accounted for less than 5% of the thematic network, indicating limited representation.

**Conclusion:**

Despite sustained scientific activity, the quantitative structure of the literature indicating persistent asymmetries in thematic representation. Notably, psychosocial outcomes, assistive technology applications, and the integration of functional and metabolic perspectives remain underexplored. These findings highlight clear research gaps and underscore the need for more interdisciplinary approaches and broader geographic participation to advance evidence-based exercise interventions for individuals with SCI.

## 1 Introduction

Spinal cord injury (SCI) is a complex neurological condition associated with persistent impairments in motor, sensory, and autonomic functions, leading to substantial functional limitations and reduced quality of life. Exercise-based rehabilitation has consistently demonstrated benefits across multiple domains, including physical performance, cardiorespiratory function, neural plasticity, and psychosocial well-being, positioning physical exercise as a core therapeutic strategy in SCI management (1–6).

Parallel to clinical advances, scientific output related to SCI rehabilitation has expanded markedly over the past two decades. Nevertheless, this body of literature remains methodologically heterogeneous and unevenly distributed across regions and research centers. Previous bibliometric studies have explored general trends in SCI research or rehabilitation more broadly, reporting growth patterns, emerging themes, and collaborative networks at a macro level (7–10), However, these analyses did not specifically isolate physical exercise as a distinct research domain, nor did they integrate data from multiple bibliographic databases to comprehensively capture the interdisciplinary nature of exercise-related SCI research.

Physical exercise research in SCI spans diverse scientific domains, including physiology, biomechanics, clinical rehabilitation, engineering, and behavioral sciences, resulting in dispersed publication patterns across journals and databases. Therefore, a dedicated scientometric assessment is warranted to accurately map its conceptual structure, thematic evolution, and collaboration networks. The integration of Web of Science, PubMed, and Scopus enables broader coverage of biomedical, rehabilitation-focused, and multidisciplinary sources, reducing database-specific bias and enhancing the robustness of the analysis.

Scientometric methods provide a systematic framework for quantifying the evolution and structure of scientific knowledge by examining publication trends, citation patterns, and relational networks among authors, institutions, and research themes (11). Applying this approach to exercise-based SCI research allows the identification of consolidated areas as well as topics with lower relative frequency and network centrality that may not be evident through traditional narrative or systematic reviews. To address the aforementioned gap is required a comprehensive assessment of how research on exercise and SCI has evolved globally. Therefore, this study conducted a scientometric analysis of the literature on physical exercise and SCI, examining temporal trends, influential sources, leading authors and institutions, collaboration networks, and the conceptual structure that characterizes the field. Accordingly, the specific aims of this scientometric study were to: (i) map the temporal evolution and productivity of scientific publications on physical exercise and spinal cord injury; (ii) identify leading journals, authors, institutions, and countries through collaboration and citation networks; and (iii) analyze the conceptual and thematic structure of the field using keyword co-occurrence and multiple correspondence analysis.

## 2 Methods

This is a scientometric study that quantitatively evaluates scientific output on a specific topic through the analysis of original research articles, allowing for the mapping of its evolution and the identification of trends in the literature (11).

### 2.1 Data collection

The databases Web of Science (WoS) (https://www.webofscience.com), PubMed (https://pubmed.ncbi.nlm.nih.gov), and Scopus (https://www.scopus.com/home.uri) were searched for articles published between 2004 and 2024. This time frame was chosen because it encompasses the last two decades of research on the topic, allowing the identification of publication trends, methodological advances, and patterns of scientific collaboration. These databases were selected for their broad scientific coverage and thematic complementarity. Their combined use enables the retrieval of both biomedical literature and multidisciplinary studies, increasing the sensitivity of the search and reducing potential biases in record identification.

The search strategy was constructed with the inclusion of central terms to increase the number of potential studies on the topic and reduce the risk of premature exclusion of relevant studies.

Therefore, the string was structured into three complementary conceptual blocks:

population/condition: wheelchair; wheelchair users; people with disabilities; paraplegia; tetraplegia; quadriplegia; spinal cord injury; lower limb paralysis; physical disability; wheelchair athletes; Paralympic athletes; wheelchair sports; adapted sports.

Intervention: exercise; physical exertion; aerobic exercise; ergonomics; cycle ergometer; arm cycle ergometer.

Outcomes: physical fitness; cardiorespiratory endurance test; cardiopulmonary exercise test; cardiorespiratory fitness; oxygen consumption; aerobic capacity; VO_2_ max.

This strategy has reached interdisciplinary fields, which use different terminologies to describe similar populations, interventions, and outcomes.

A standardized and unified search string was developed using boolean operators to combine descriptors directly related to the central theme of the study. The construction of the search strategy was based on descriptors identified in the titles, abstracts, and keywords of previously indexed publications in the field. Different combinations of these terms were tested, and the string that provided the greatest coverage and accuracy in record retrieval was adopted as the final search strategy. Data collection was carried out on the bibliographic platforms PubMed, Scopus, and Web of Science, using the same standardized search string to ensure comparability across sources. To ensure methodological consistency between databases, the field tags used on each platform were standardized. Thus, “Title/Abstract” was used in PubMed, the “TITLE-ABS-KEY” field in Scopus, and the “TS = Topic” field in Web of Science, as they are semantically equivalent and allow for the harmonized retrieval of titles, abstracts, and keywords. Time filters were applied to cover the period from January 1, 2004 (oldest study evaluated—April 1, 2004) to December 31, 2024 (most recent study evaluated—September 2, 2024). Original articles with observational, clinical, and experimental designs were included, involving cross-sectional and longitudinal studies, clinical trials, quasi-experiments, and experiments conducted with humans or animal models, regardless of language.

Grey literature was excluded, since scientometric reviews depend on standardized metadata, systematic indexing, and comparable metrics, conditions that are generally not met by this type of document.

The results were exported in database-specific formats, generating the following files: WebofScience.bib, PubMed.txt, and Scopus.bib, which were subsequently merged into a single database for analysis. The files were downloaded on June 10, 2025. Table 1 presents the details of each platform consulted, including the standardized and reproducible search string, the platform used, and the file format exported.

**Table 1:** Description of search strings, searched fields, and export formats of bibliographic platforms.

Standardized search	Database	File format
(“wheelchair” OR “wheelchair users” OR “wheelchair-bound” OR “wheelchair dependent” OR “wheelchair athletes” OR “paralympic athletes” OR “adapted sports” OR “wheelchair sports” OR paraplegia OR tetraplegia OR quadriplegia OR “spinal cord injury” OR “lower extremity paralysis” OR “physical disabilit*” OR “people with disabilities” OR “persons with disability” OR “mobility impairment” OR “neurological impairment”) AND (“exercise” OR “aerobic exercise” OR “physical exertion” OR “exercise tolerance” OR “cycle ergometer” OR “arm ergometer” OR “arm crank ergometer” OR “upper-body exercise” OR “wheelchair propulsion test” OR ergonom*) AND (“physical fitness” OR “aerobic capacity” OR “aerobic power” OR “cardiorespiratory fitness” OR “cardiopulmonary exercise test” OR “submaximal test” OR “oxygen consumption” OR “VO2 max” OR “VO2max” OR “VO2peak” OR “maximal aerobic capacity” OR “cardiorespiratory assessment”)	WOS	BIB
	PubMed	TXT
	Scopus	BIB

The data retrieved from the three databases were merged using the Bibliometrix 4.1.3 package (12) in R version 4.4.2 (R Core Team, 2024). The deduplication process was performed automatically during the merging of the three databases by the RStudio software, using the internal functions of the Bibliometrix package, which identify duplicate records based on combinations of DOI, title, authors, and year of publication. This procedure ensured the removal of duplicates, guaranteeing the uniqueness of the records analyzed. The screening process was conducted in three sequential stages to ensure methodological reproducibility. In the first stage (automated filtering) duplicates and non-original documents (reviews, meta-analyses, editorials, letters, and conference abstracts, and gray literature) were automatically excluded using Bibliometrix internal functions. yielding 1,232 unique records. After automated exclusion, 1,020 original articles remained.

In the second phase (manual screening), two independent reviewers evaluated titles and abstracts to confirm scope and identify any duplicates not detected automatically. Forty-four records were excluded at this stage, resulting in 976 potentially eligible articles.

**Figure 1:**
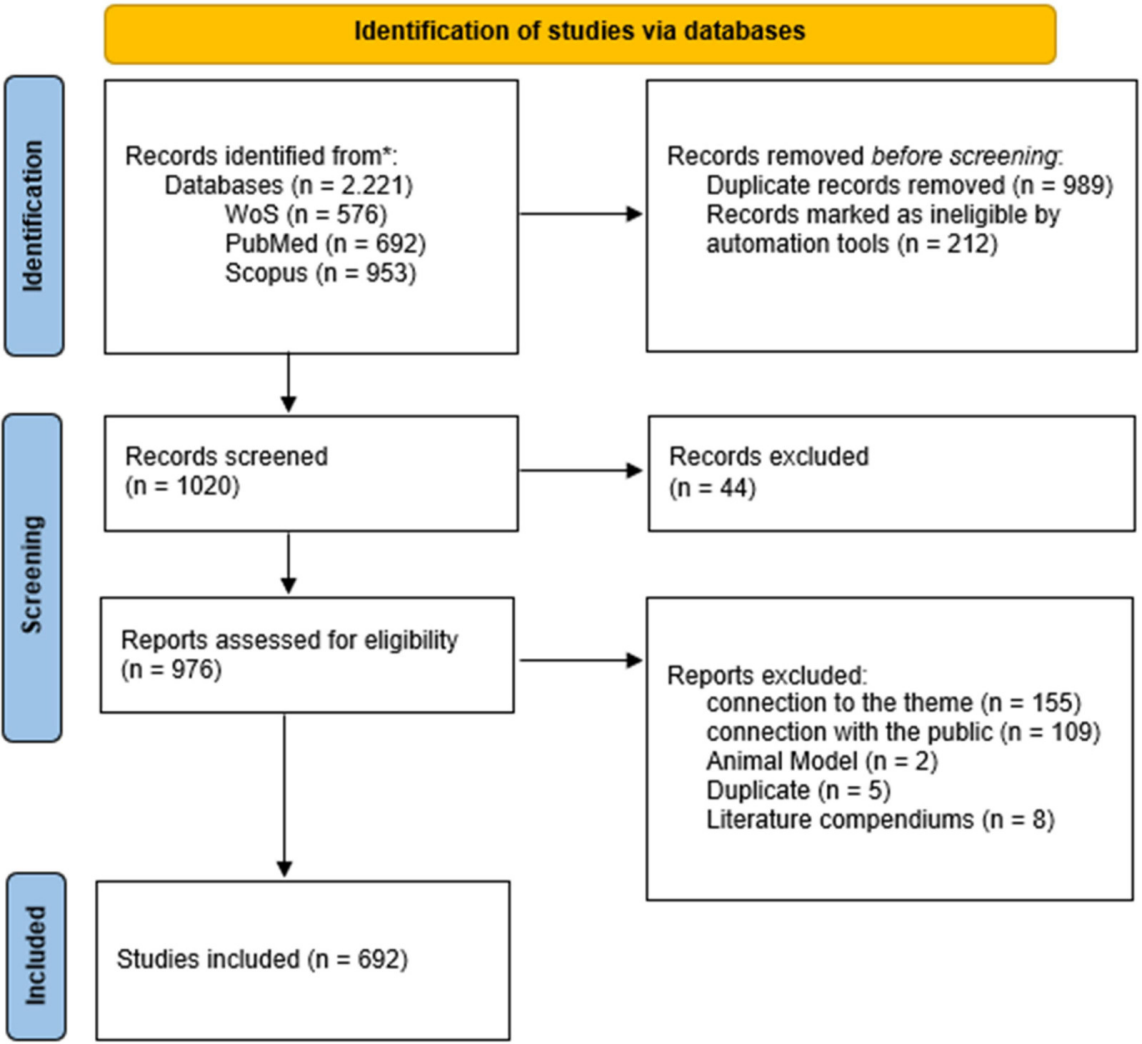
PRISMA 2020 flow diagram describing the research steps.

In the third stage (eligibility assessment), explicit inclusion criteria based on an adapted PICO strategy were applied. Eligible studies were original articles that: (i) included human participants with spinal cord injury (Population); (ii) implemented or evaluated structured physical exercise interventions (Intervention); and (iii) reported outcomes related to functional capacity, physical performance, or health parameters (Outcomes). Studies not aligned with the topic (*n* = 154), without the target population (*n* = 109), animal models (*n* = 2), remaining duplicates (*n* = 5), and collections of studies (*n* = 8) were excluded. A total of 692 articles were retained for analysis (Figure 1 shows the steps for screening the references using PRISMA flow diagram).

Before the final analysis, steps were taken to correct metadata, inconsistent identifiers (DOI), and standardize author and journal names, with the aim of reducing spelling variations and ensuring greater accuracy in the analyses.

### 2.2 Scientometric analyses

Descriptive and mapping-based scientometric analyses were conducted using the Bibliometrix package (version v4.1.3) implemented in the R environment (version 4.4.2) (12), VOSviewer software (version 1.6.19) (13), and Microsoft Excel® as a complementary tool for data organization and visualization.

**Figure 2:**
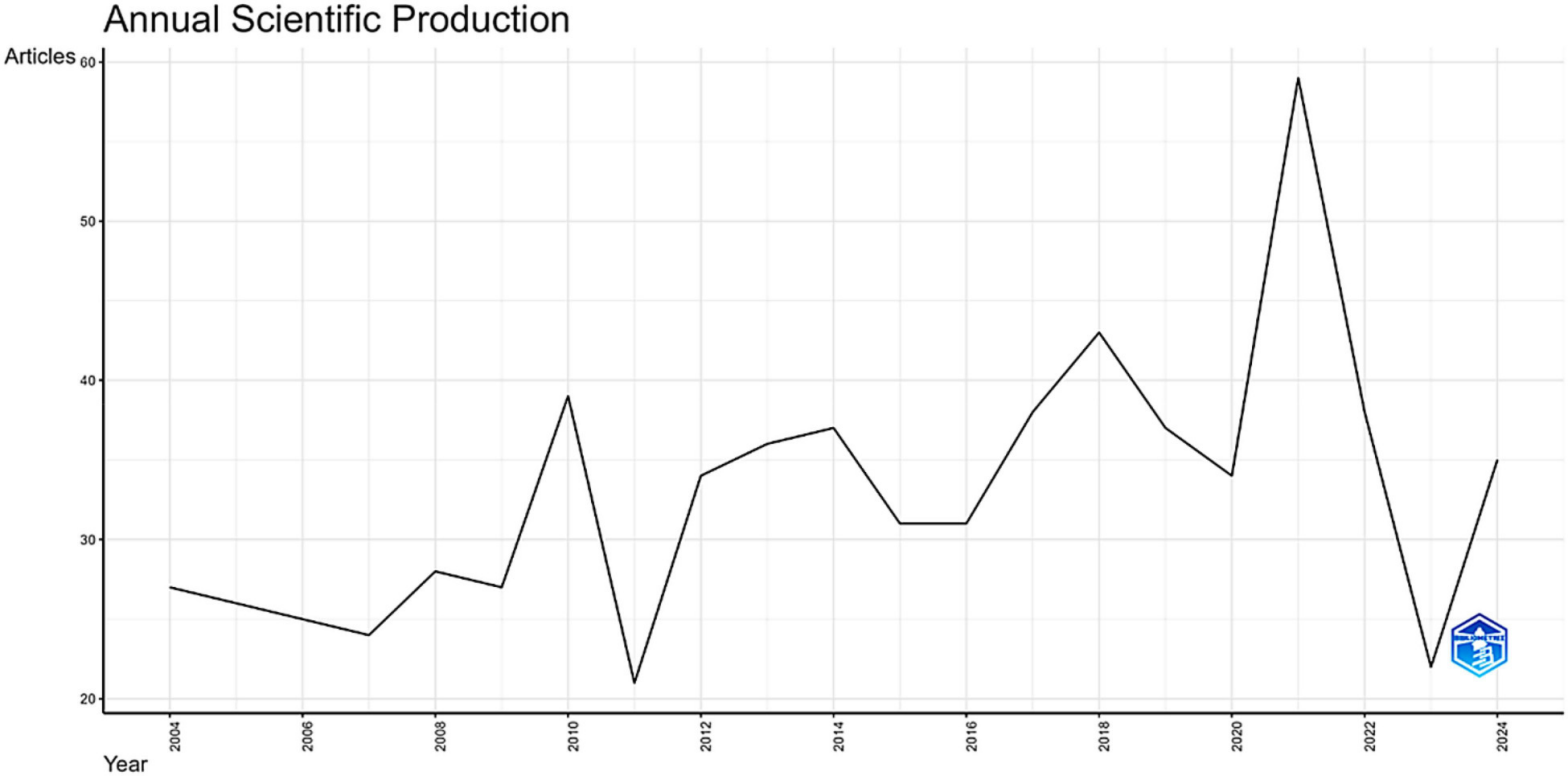
Temporal trend of publications on exercise and spinal cord injury. This figure displays the annual number of publications related to physical exercise (PE) and spinal cord injury (SCI) indexed in Web of Science, Scopus, and PubMed. The curve illustrates the overall growth trajectory, with fluctuations highlighting peaks and declines in scientific output. Node size corresponds to the number of publications per year. The data regarding the number of publications, as well as the themes identified in each period (frequency), are presented in Supplementary Table 1.

**Figure 3:**
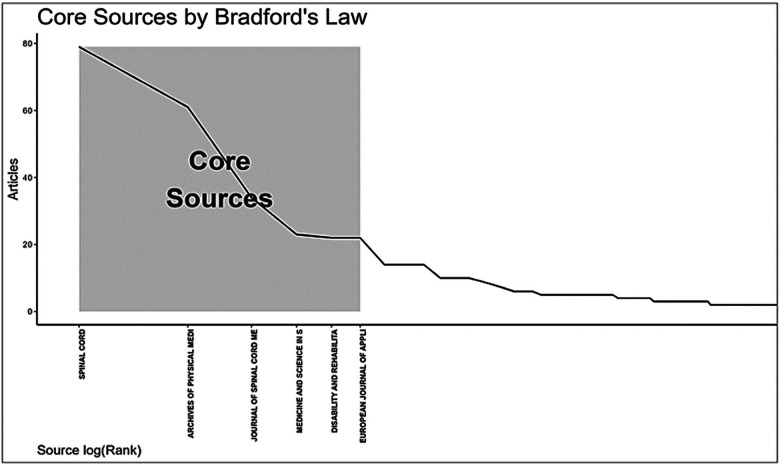
*Bradford's Law of scattering (1934).* The figure depicts the rank–frequency distribution of journals according to publication volume. The shaded “core zone” identifies the seven journals that concentrate the largest share of articles on PE and SCI. Each bar represents one journal; height = number of publications; cumulative curve = cumulative percentage of total output.

**Figure 4:**
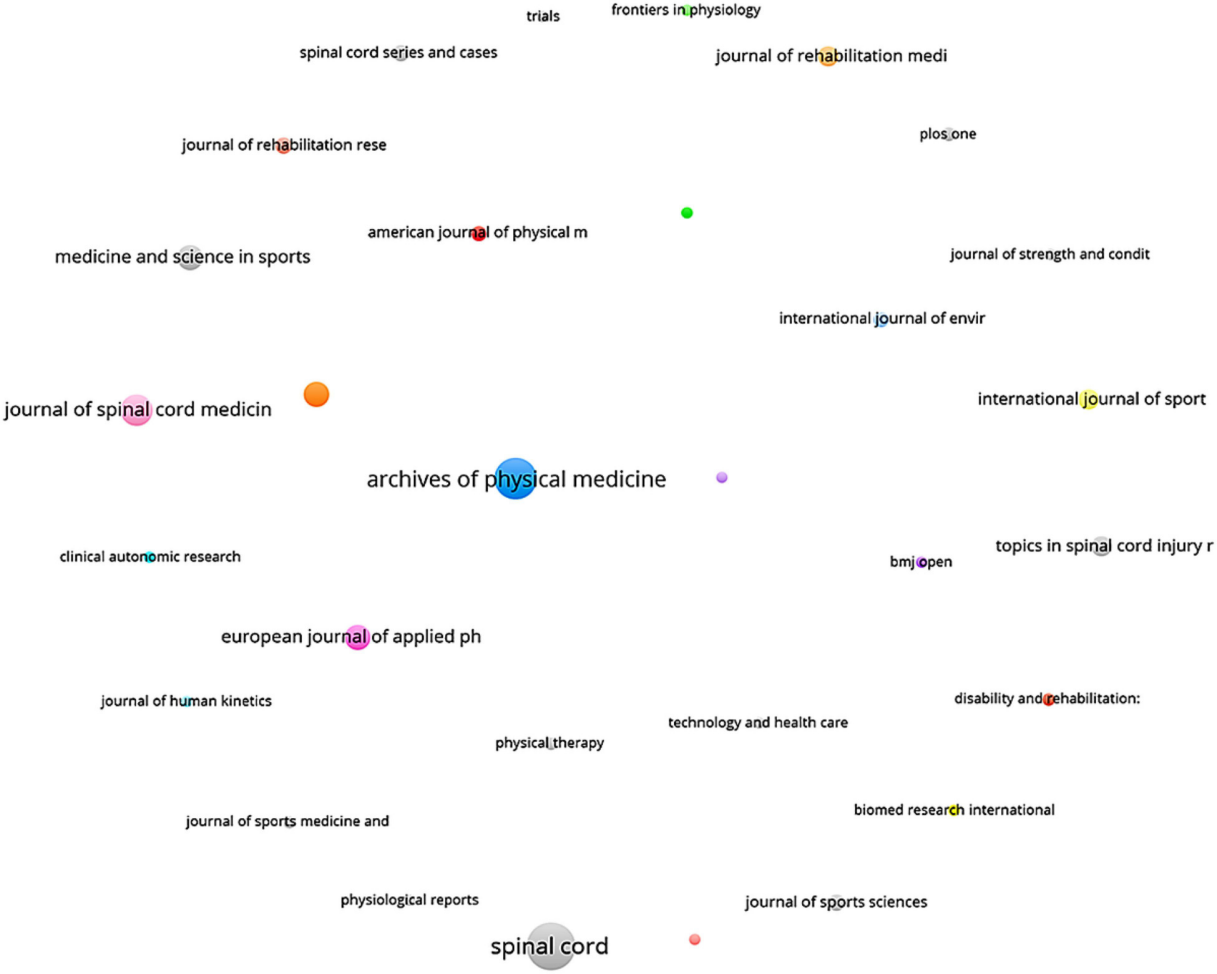
Scientific production over time on exercise and spinal cord injury. This figure depicts the temporal distribution of scientific output among the most productive authors in the field from 2004 to 2024. Each node represents an author in a given year; node size is proportional to the number of publications in that year, while color intensity reflects annual citation impact (total citations per year). This visualization allows the simultaneous assessment of productivity and citation influence over time. The figure highlights sustained contributions by a limited number of authors, consistent with the low-density and hub-centered structure observed in the collaboration network. Quantitative productivity and citation metrics are detailed in Supplementary Tables S2, S3.

**Figure 5:**
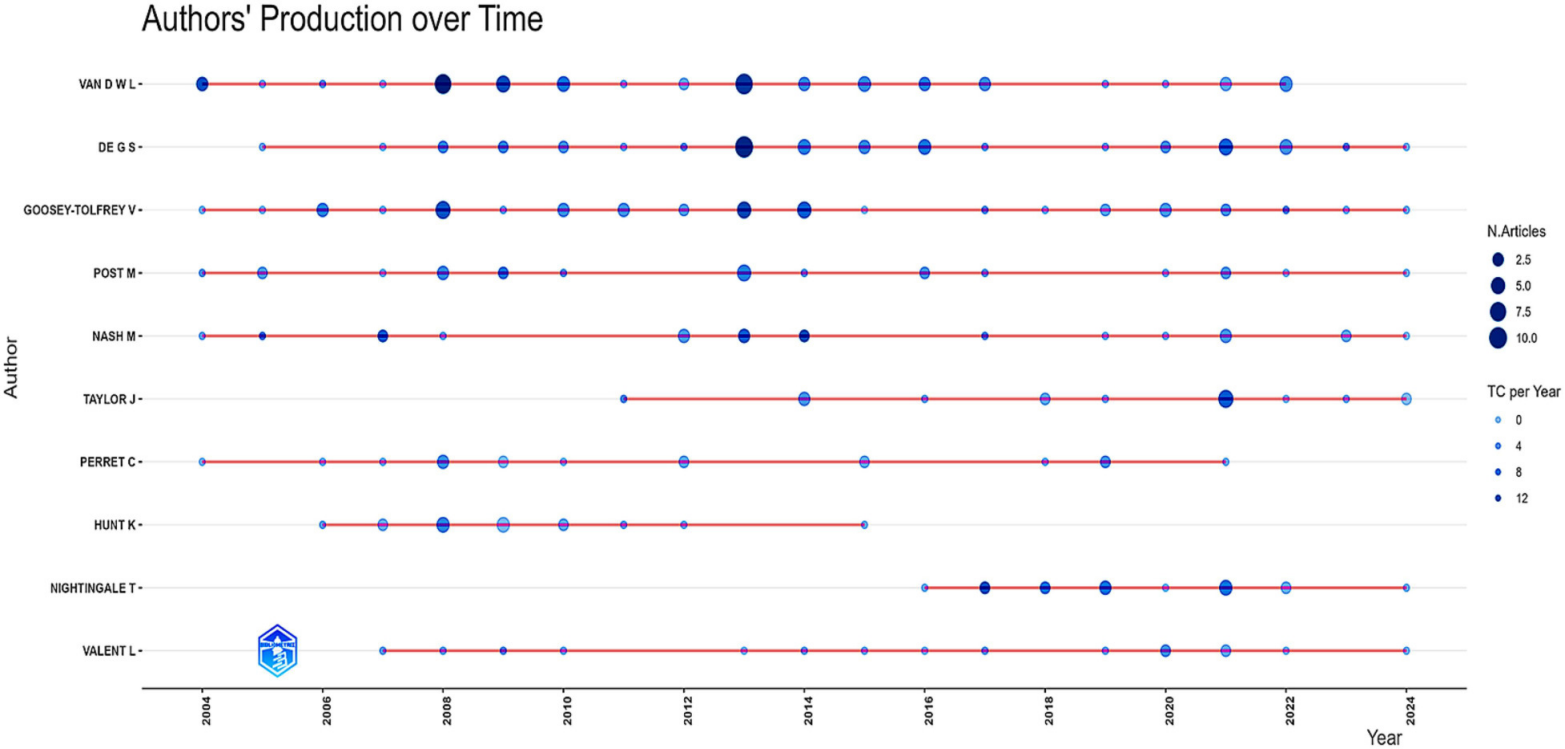
Scientific collaboration network Among authors. This figure illustrates the scientific collaboration network among authors based on co-authorship relationships. Nodes represent authors, with node size proportional to total publication output. Edges indicate co-authorship relationships, with thickness proportional to the number of shared publications. Node proximity reflects collaboration frequency. Colors represent clusters identified using the VOSviewer modularity-based algorithm. Only authors with a minimum of two publications were included in the network. Quantitative network metrics (PageRank, betweenness, closeness, and total link strength) are reported in Supplementary Tables S2–S4.

**Figure 6:**
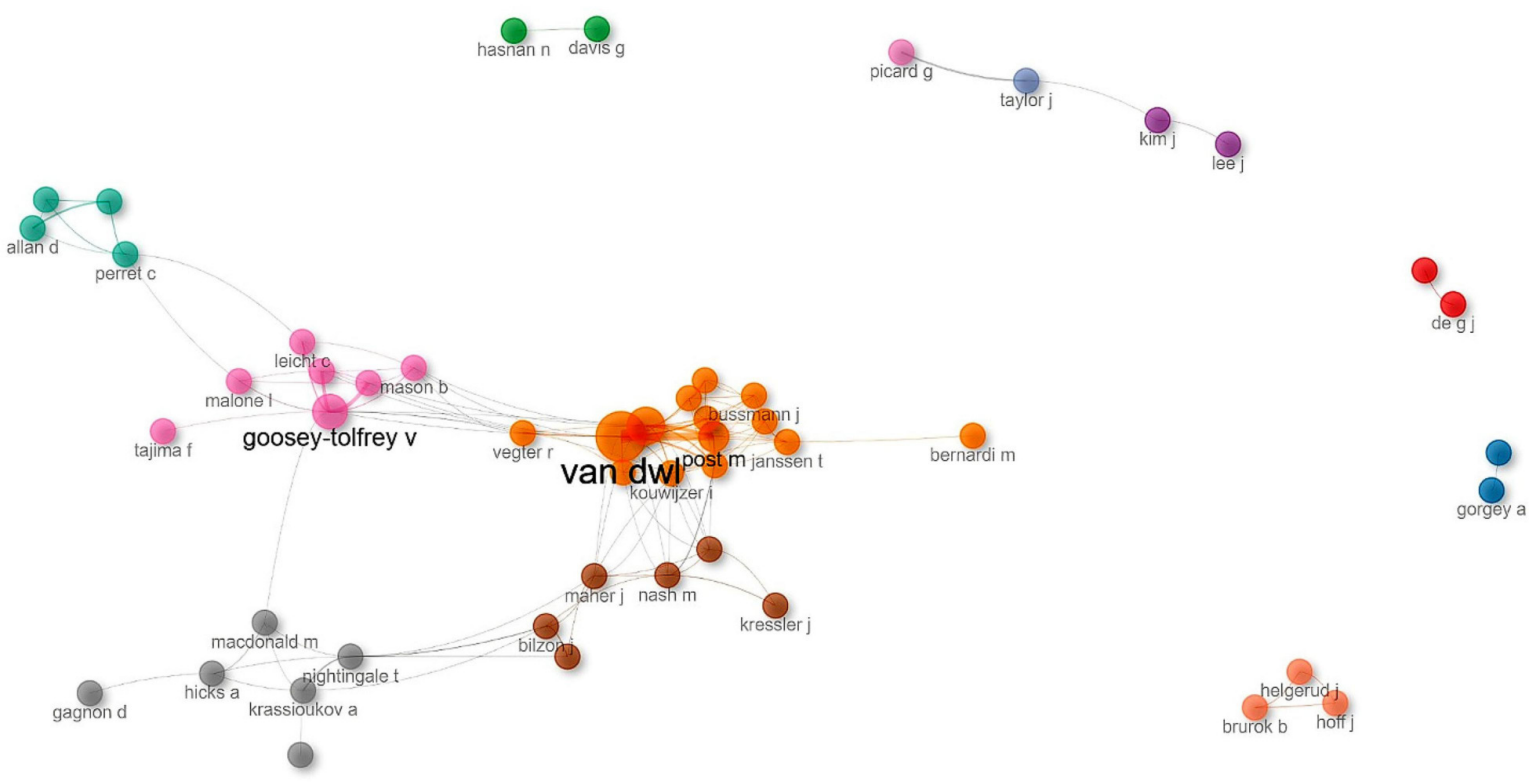
Trending topics in scientific production on physical exercise and spinal cord injury (2004–2024). This timeline shows the evolution of author keywords over time. Each bubble corresponds to a keyword; bubble size represents occurrence frequency; color indicates the average year of appearance. Data regarding trending topics in scientific production on physical exercise and spinal cord injury, by frequency and year, can be found in Supplementary Table S5.

**Figure 7:**
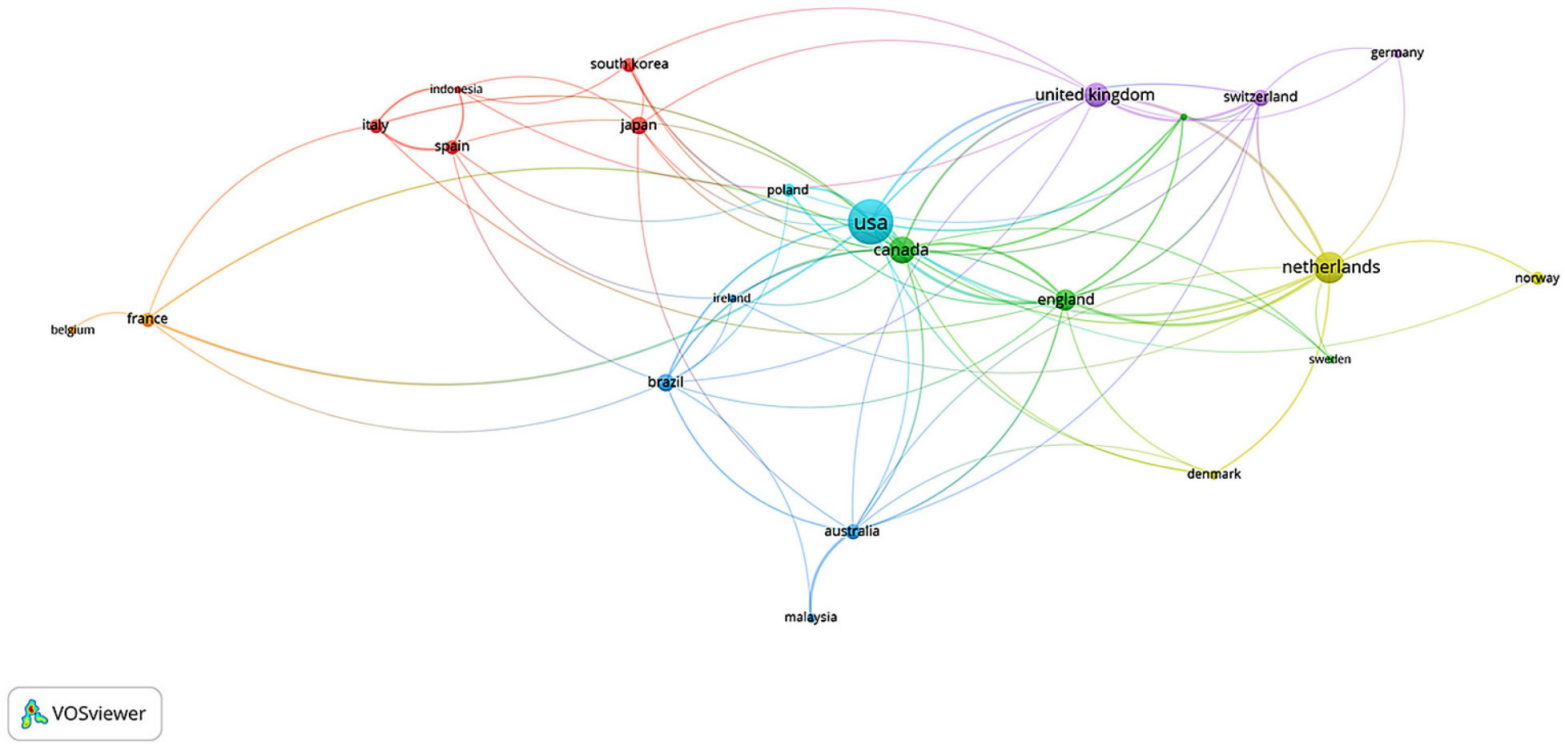
Conceptual structure map from multiple correspondence analysis (MCA). This two-dimensional map represents the conceptual structure of research on PE and SCI. Each point corresponds to a keyword; Dim 1 and Dim 2 are latent dimensions derived from MCA, capturing the principal conceptual variance in the dataset. The data regarding the conceptual regions present in the MCA, such as the number of terms, percentages, and thematic synthesis, can be observed in supplementary Tables S8 and S9.

Using the Bibliometrix package (12), the following analyses were performed: general characterization of the dataset (document type, language, year of publication, keywords, and authors); temporal evolution of scientific production (Figure 2); identification of the most productive journals based on Bradford's Law (Figure 3); authors' productivity over time (Figure 4); co-authorship networks among authors (Figure 5); identification of emerging research topics by publication period (Figure 6); and mapping of the conceptual structure based on Multiple Correspondence Analysis (MCA). MCA was selected as an appropriate multivariate technique for categorical data, enabling the identification of patterns of association and semantic proximity among the main themes investigated. The selection and treatment of terms were performed automatically by Bibliometrix, which applies internal frequency and co-occurrence criteria for the construction of the factorial space, without the application of additional manual filters (Figure 7).

The following analyses were conducted using VOSviewer.

Co-citation and co-authorship networks were normalized using the association strength method, and clustering resolution and layout parameters were maintained at the default settings.

**Figure 8:**
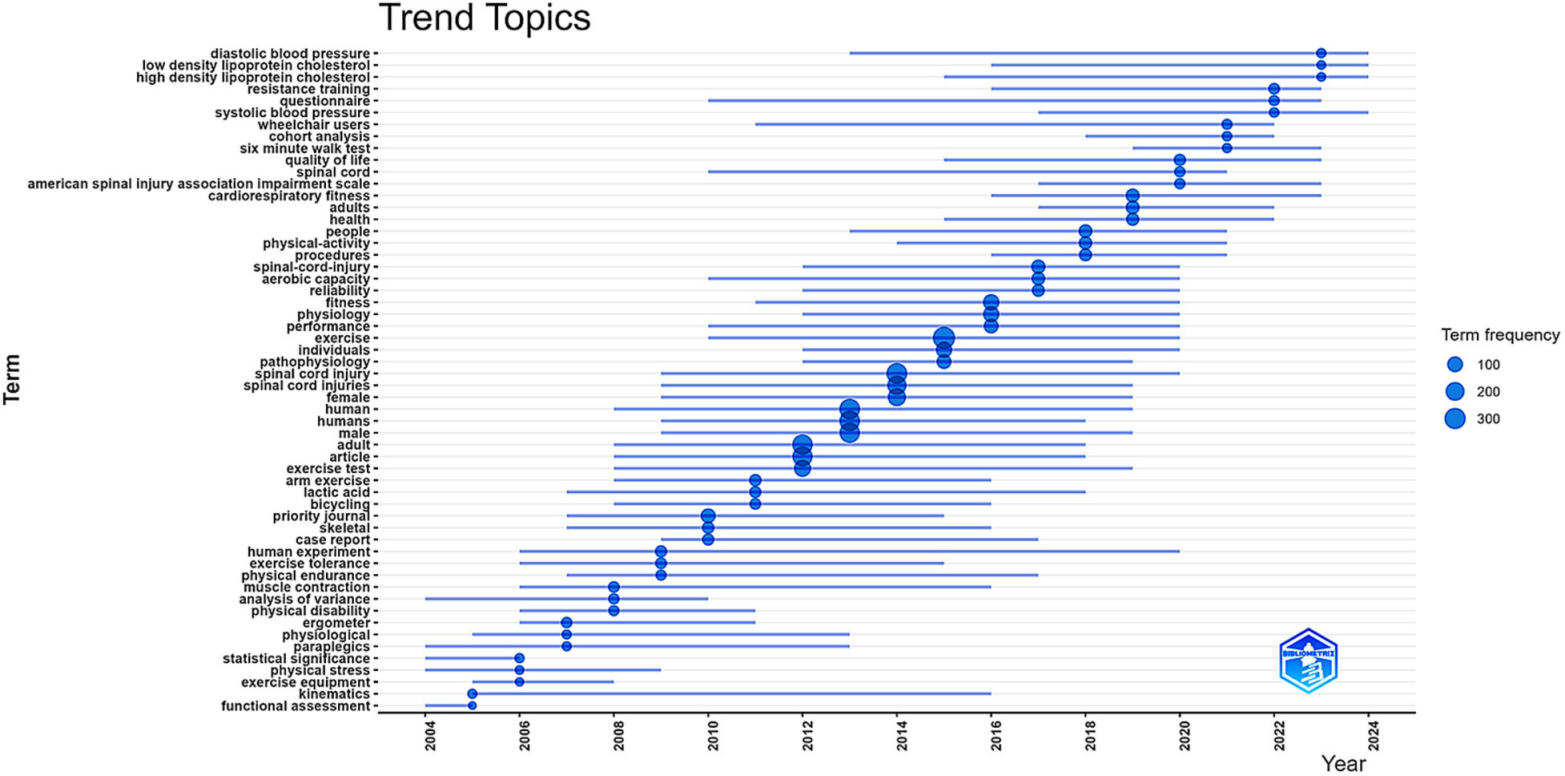
Journal co-citation network. Nodes represent journals; node size indicates total citation count; edge thickness denotes the strength of co-citation (frequency with which two journals are cited together); and colors indicate clusters formed by the VOSviewer modularity algorithm. The blue cluster (e.g., Archives of Physical Medicine and Rehabilitation) dominates in citation volume, followed by gray (Spinal Cord) and pink (Journal of Spinal Cord Medicine).

For the journal co-citation network, a minimum threshold of five documents per journal was applied for inclusion in the analysis. The software calculated the total link strength between sources and selected the 30 journals with the highest co-citation intensity, enabling the identification of the most influential and structurally central journals in the analyzed literature (Figure 8).

**Figure 9:**
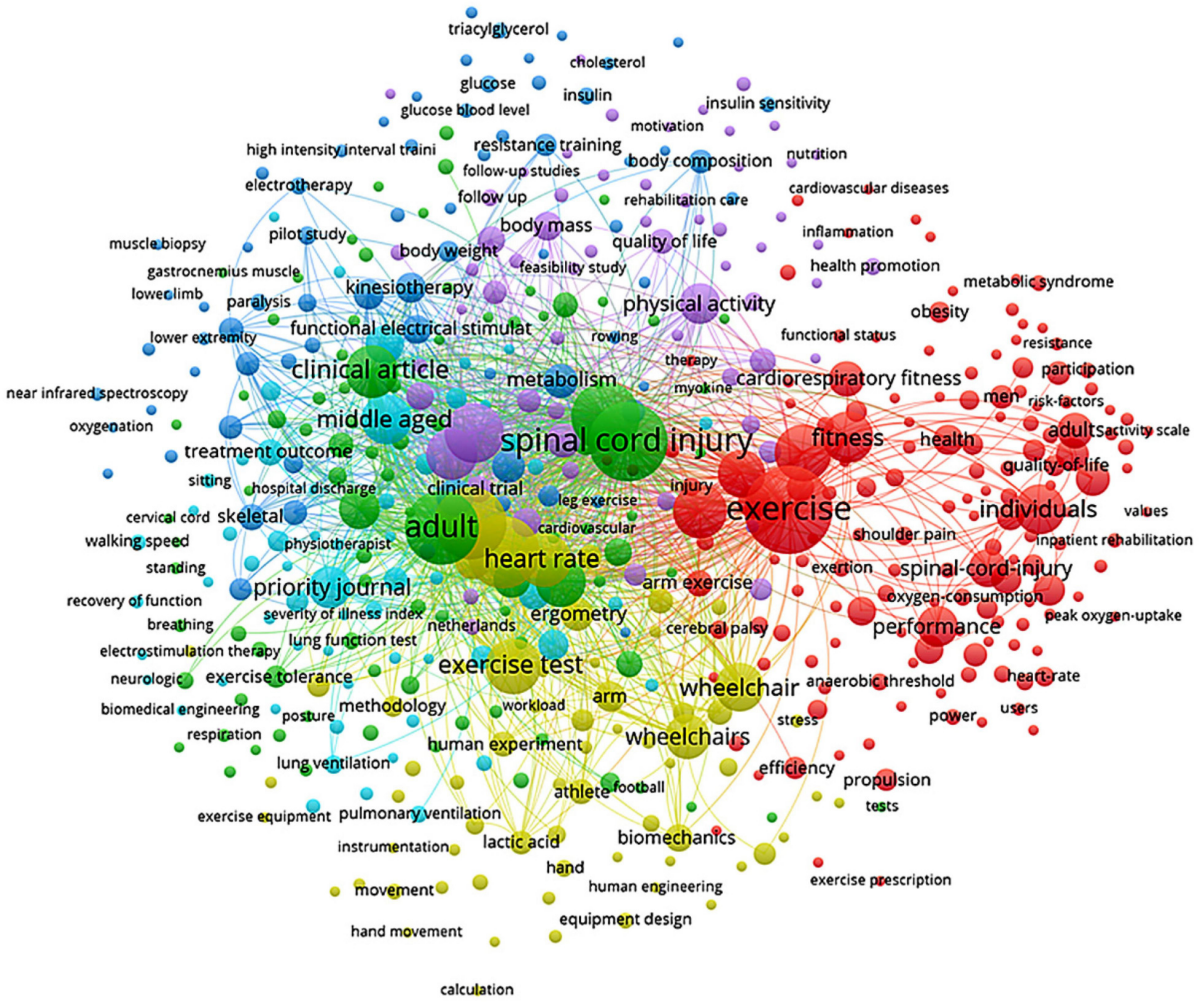
Network of collaboration between countries in scientific production on physical exercise and spinal cord injury. Nodes represent countries, with node size proportional to total publication output. Edges indicate international co-authorship links, with thickness proportional to collaboration frequency (total link strength). Node proximity reflects the intensity of collaborative interactions. Colors represent regional clusters identified through modularity-based community detection. Only countries with at least three publications were included. Quantitative indicators are detailed in Supplementary Table S4.

For the international collaboration network, a maximum limit of 25 countries per document was applied to minimize distortions caused by large multicenter consortia. Only countries with at least five published documents were included, and no minimum citation threshold was applied (Figure 9).

**Figure 10:**
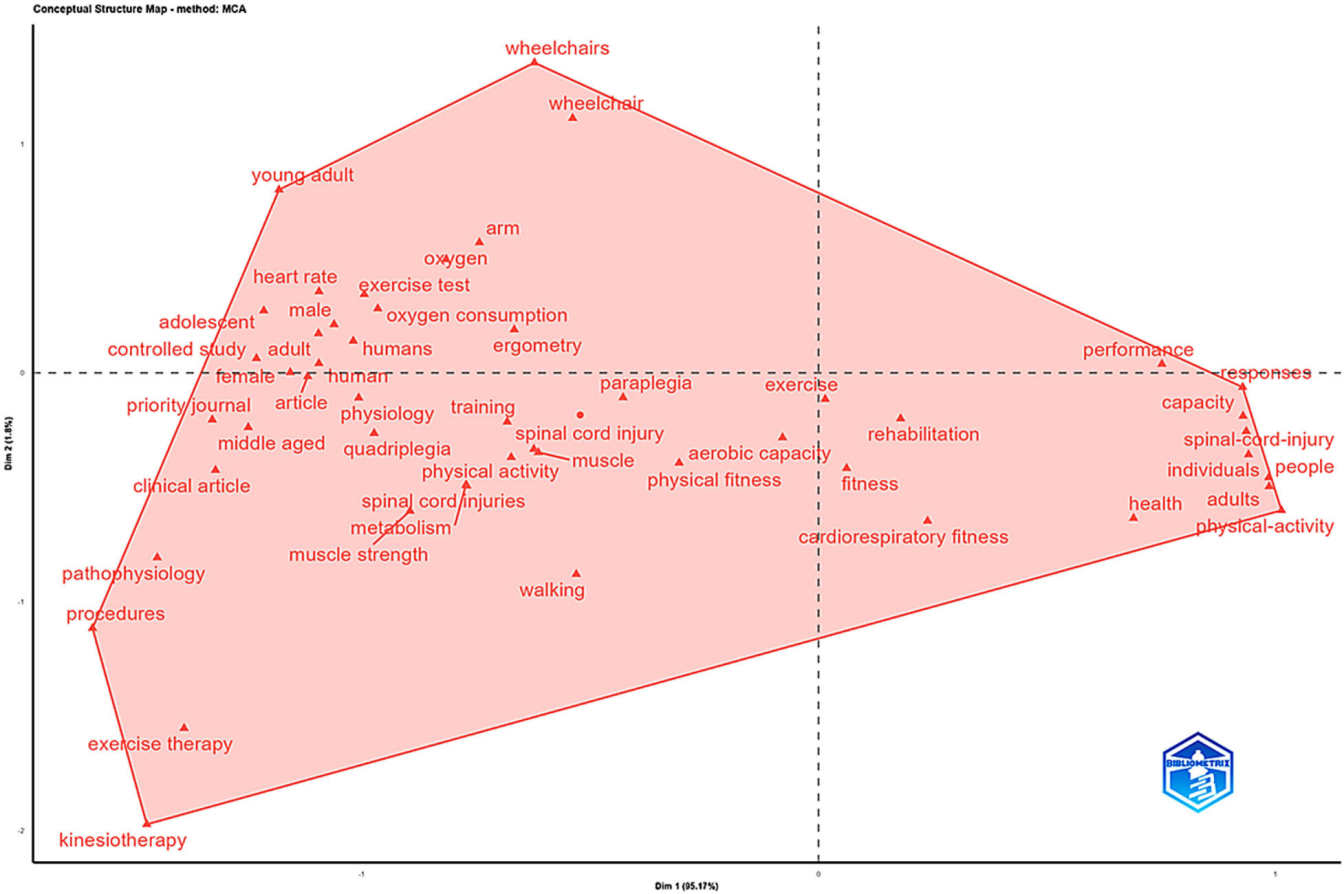
Co-occurrence network of author keywords on physical exercise and spinal cord injury. The map shows the thematic organization of the literature, with colored clusters grouping frequently associated terms. Nodes represent keywords, with size proportional to frequency of occurrence. Edges indicate co-occurrence strength, with thicker links reflecting higher co-occurrence frequency. Colors represent thematic clusters identified using the VOS algorithm. Only keywords with a minimum occurrence of five were included. Percentage distributions and absolute frequencies are provided in Supplementary Tables S6 and S7.

For the authors' keyword co-occurrence network, author keywords were used as the unit of analysis, applying the full counting method and a minimum threshold of five occurrences per keyword. Of the 4,340 keywords identified, 507 met this criterion and were automatically included in the construction of the network clusters, allowing the identification of thematic patterns and the conceptual structure of the scientific field (Figure 10). A minimum threshold of five keyword occurrences was adopted to ensure analytical robustness while reducing noise from sporadically used terms, a criterion commonly applied in keyword co-occurrence analyses.

Microsoft Excel® was used to analyze the most globally cited articles (Table 1), including calculations of total citation count, annual citation average, and normalized citation index. Excel was also used to organize data on institutional productivity (Table 2), facilitating the ranking of universities with the highest scientific output.

**Table 2:** Ranking of the most globally cited documents on physical exercise and spinal cord injury.

Ranking	Author (Year)	Total citations	Citations/Year	Normalized TC	DOI
1	Scekza W, 2005	194	9.24	4.48	10.1097/01.phm.0000171172.96290.67
2	Griffin L, 2009	192	11.29	7.41	10.1016/j.jelekin.2008.03.002
3	Ryan T, 2012	164	11.71	6.97	10.1152/japplphysiol.00319.2012
4	Nash M, 2005	152	7.24	3.51	10.1097/01.NPT.0000282514.94093.c6
5	Cramer S, 2007	147	7.74	4.31	10.1007/s00221-006-0662-9
6	Nash M, 2007	131	6.89	3.84	10.1016/j.apmr.2006.10.003
7	Collins E, 2010	125	7.81	4.89	10.1249/MSS.0b013e3181bb902f
8	Manns P, 2005	123	5.86	2.84	10.1016/j.apmr.2004.11.020
9	Anneken V, 2010	110	6.88	4.3	10.1038/sc.2009.137
10	Evans N, 2015	108	9.82	6.02	10.1310/sci2102-122

Normalized TC—Normalized Total Citations were calculated by dividing the total number of citations of each article by its publication age (years since publication). This adjustment reduces the bias favoring older publications and allows a more balanced comparison of scientific impact across different publication years. DOI, digital object identifier.

## 3 Results and discussion

The analysis covered the period from 2004 to 2024, resulting in a total of 692 documents distributed across 214 publication sources. The average annual growth rate in scientific output over this period was 1.31%. The document set has a mean publication age of 10.2 years, indicating that a substantial portion of the literature is well-established and has been consistently cited within the field. The average number of citations per document was 18.4. A total of 2,257 unique authors were identified, of whom 11 published as sole authors. The average number of co-authors per article was 5.48, and the rate of international collaboration reached 11.99%. Regarding the textual content of the documents, 2,848 terms were indexed as keywords, and 1,293 author-provided keywords were identified.

### 3.1 Temporal evolution of scientific production

Figure 2 presents the annual scientific production on PE and SCI from 2004 to 2024. Over this 20-year interval, the field exhibited a stable publication pattern with modest year-to-year variability, with annual outputs generally ranging between 23 and 39 articles. Across the full period, this corresponds to an average of approximately 33 articles per year and an average annual growth rate of 1.31%, indicating gradual expansion rather than exponential growth. These patterns are consistent with previous mapping studies showing that SCI rehabilitation and related subfields tend to maintain steady and moderate publication volumes over time (7–10), positioning PE and SCI as a consolidated research area within the rehabilitation and neuroscience literature (1, 2, 4, 9).

The highest annual output was observed between 2020 and 2021 (*n* = 59). From a quantitative perspective, this value represents a transient deviation above the historical range rather than a structural breakpoint in the time series, as it remains within the same order of magnitude observed throughout the study period. Given the descriptive nature of the scientometric design and the absence of keyword-level or topic-shift analyses, no causal inference is made regarding the drivers of this peak, which is therefore interpreted strictly as a short-term amplification in publication volume.

Following the 2021 peak, publication output declined by approximately 62.7% between 2021 and 2023, returning to levels comparable to those observed in earlier years. This reduction is consistent with a regression toward the long-term baseline rather than evidence of sustained contraction. Within the context of the complete time series, fluctuations of this magnitude fall within the expected variability of a field characterized by stable and moderate production. Preliminary data for 2024 indicate a slight rebound; however, due to indexing delays and incomplete database coverage for the most recent year, these values are interpreted cautiously. Overall, the temporal profile depicts a field marked by long-term stability with superimposed short-term fluctuations, including one pronounced peak followed by normalization toward historical levels, rather than abrupt expansion or decline.

### 3.2 Leading journals and most globally cited articles

Figure 3 presents the journals ranked by productivity on the topic. The shaded area highlights the core sources, which concentrate the majority of publications. A strong concentration is observed in seven core journals, indicating concentration of publications within a limited set of core journals. This pattern reflects a preference within the scientific community for a select group of journals that structure the academic discourse in this area (7, 8).

Among these, the journal *Spinal Cord*, published by the Nature Publishing Group, stands out for its international circulation. According to the *Journal Citation Reports (JCR)*, it holds an impact factor of 2.2 (Q2 in Clinical Neurology and Rehabilitation), a Scopus CiteScore of 3.5 (2023), an H-index of 131, and a 2024 SJR score of 0.945, placing it in Q1. *Spinal Cord* is considered a key publication for SCI research, covering original studies (14), systematic reviews (15), and clinical guidelines (16).

Its relevance stems from its editorial focus on SCI-related topics, encompassing clinical and surgical aspects (17), psychosocial issues (18), epidemiology (19), and technological innovations (20). This editorial direction provides visibility to research that might otherwise be dispersed across broader, generalist journals. Furthermore, *Spinal Cord* regularly publishes consensus documents (21) and international guidelines (22), serving as a key reference for clinical practice and public policy formulation aimed at motor disability rehabilitation.

Figure 8 presents the journal co-citation analysis, which identifies the main intellectual references based on journals that are frequently cited together within the sample articles. Node proximity reflects higher co-citation frequency, while colors indicate clusters generated by the VOSviewer modularity algorithm, grouping journals with strong relational patterns within the citation network (13).

The blue cluster is led by Archives of Physical Medicine and Rehabilitation which emerges as the most central journal in the co-citation network, accounting for 72 included documents and exhibiting the highest overall co-citation strength within the dataset. Its prominence is consistent with prior scientometric studies that identify this journal as a core reference in SCI rehabilitation research (9). It is published by Elsevier and focuses on physical medicine and rehabilitation, particularly physiotherapy, physical modalities, clinical interventions, and quality of life among individuals with disabilities. Its 2023 impact factor is 3.6.

Spinal Cord (gray cluster), published by Nature since 1963, a core journal in the SCI field, with an impact factor of 2.1 (2023) and approximately 807 citations within our co-citation matrix (7). Its high co-citation frequency indicates long-term integration into the intellectual base of the field, rather than short-term citation inflation.

The pink cluster includes the Journal of Spinal Cord Medicine, which occupies the third position in co-citation frequency (≈303 citations). Its placement reflects consistent cross-referencing with both clinical rehabilitation and applied exercise research, confirming its interdisciplinary role in SCI-related literature (8, 10). Medicine & Science in Sports & Exercise (green cluster), although not among the top three by absolute citation count, shows high link strength with rehabilitation-focused journals, indicating its relevance in bridging exercise science and SCI rehabilitation research (9).

Our findings align with those of previous scientometric studies. Liu et al. (9), who analyzed literature from 1997 to 2016, identified the same three journals as the most influential in the field. The persistence of this hierarchy over time supports the interpretation of a stable editorial nucleus, rather than patterns associated with short-term publication fluctuations (8, 10).

Table 2 summarises the most globally cited articles on PE and SCI. The top-ranked article is by Scelza et al. (23), which has accumulated 194 citations, a value strongly influenced by its publication age and sustained annual citation rate, reflecting its early contribution to psychosocial dimensions of exercise participation in SCI. The study by Griffin et al. (24), also ranks among the most cited, combining high total citations with one of the highest average citation rates per year (≈11 citations/year), which supports its methodological relevance in functional electrical stimulation research.

Among the top-cited documents, the article by Ryan et al. (25) shows the highest average citation rate (11.71 citations/year) indicating continued relevance over time rather than legacy effects alone. This study occupies a central position in the literature by providing standardized protocols for cardiovascular and metabolic assessment during adapted aerobic exercise, which are repeatedly cited across clinical, physiological, and methodological research streams (26, 27). Nash (28) is another highly cited source, whose citation dominance reflects its foundational role in framing cardiometabolic risk monitoring and secondary prevention in individuals with SCI, themes that cut across multiple research clusters.

The article by Cramer et al. (29) also appears prominently in normalized citation metrics, reflecting both its citation rate relative to publication age and its cross-cluster relevance, as it integrates neurophysiological mechanisms with rehabilitation and exercise-based interventions. Its frequent citation in subsequent reviews and meta-analyses (30–35). supports its position as a conceptual bridge between neuroscience and applied rehabilitation research. Together, these documents demonstrate that high citation impact in PE and SCI research is primarily associated with methodological centrality, cross-disciplinary applicability, and sustained relevance, rather than thematic popularity alone.

The study by Ryan et al. (25), published in the *Journal of Applied Physiology*, reported the highest average number of citations per year among the top five articles (11.71 a metric that, together with its high normalized citation index, indicates sustained relevance over time rather than citation accumulation driven solely by publication age. The study investigated cardiovascular responses to physical exercise in individuals with SCI, emphasizing safety, exercise intensity, and effort thresholds in this population. Its citation prominence is primarily explained by its methodological centrality, as it contributed to the standardization of submaximal assessment protocols and the validation of adapted peak V˙O_2_ measurements, which are recurrently cited across clinical, physiological, and rehabilitation-focused studies. These methodological features provided a technical foundation for clinical guidelines (36) and exercise prescription practices across various rehabilitation contexts (26, 27). Despite its impact, the study's focus on individuals with incomplete thoracic injuries constrains its external validity, a limitation that does not diminish its structural role within the citation network but should be considered when extrapolating findings to more severe injury profiles.

In turn, the study by Nash (28), published in the *Journal of Neurologic Physical Therapy*, achieved high citation impact due to its cross-cluster relevance, addressing cardiovascular risk and secondary prevention in individuals with SCI. It examined metabolic and hemodynamic profiles and advocated for continuous monitoring of parameters such as blood glucose, blood pressure, and dyslipidemia. Its dominance in citation metrics reflects its integrative positioning at the intersection of clinical rehabilitation, exercise physiology, and public health, rather than the strength of experimental design alone. As the study was based on observational data without randomized controlled trials, its conclusions are primarily conceptual and framework-oriented, which helps explain its frequent citation in guideline-oriented and policy-related literature, despite inherent methodological constraints.

Lastly, the study by Cramer et al. (29), published in *Experimental Brain Research*, stands out in normalized citation metrics due to its conceptual and translational centrality, integrating neurophysiological mechanisms with rehabilitation science. By addressing exercise-induced neuroplasticity, cortical reorganization, and functional recovery, the study occupies a bridging position between neuroscience and applied rehabilitation clusters, which explains its recurrent citation across thematic domains. Its influence is further evidenced by frequent inclusion in narrative reviews (30, 31, 37) and meta-analyses (32–34). Nevertheless, its reliance on preclinical or non-randomized designs limits direct clinical generalization, reinforcing the need for controlled trials to translate these mechanisms into standardized therapeutic protocols.

In summary, these highly cited studies represent complementary milestones within the citation structure of SCI rehabilitation research, with impact driven predominantly by methodological centrality, cross-disciplinary applicability, and sustained citation rates, rather than by thematic popularity alone. While each contributes to advancing psychosocial, cardiovascular, neuromotor, and adaptive dimensions of exercise in SCI, persistent methodological limitations and underexplored translational pathways highlight the need for future interdisciplinary research grounded in robust experimental designs (35).

### 3.3 Author productivity over time and scientific collaboration networks among institutions and countries

The author collaboration network is characterized by low overall density and high modularity, indicating a structure composed of well-defined collaborative communities with limited inter-cluster connectivity. Quantitative network analysis revealed a sparse global structure, in which a small number of authors exhibited high structural centrality, while most occupied peripheral positions. Centrality metrics, including PageRank, betweenness centrality, closeness centrality, and total link strength, were calculated to summarize authors' structural influence within the network (Supplementary Tables S2–S4).

Figure 4 presents the temporal distribution of scientific output among the most productive authors in the field. In this visualization, node size represents the number of publications per year, while color intensity reflects annual citation impact (total citations per year—TC/year), allowing simultaneous interpretation of productivity and influence over time. The distribution highlights a concentration of sustained productivity among a limited number of authors, consistent with the low-density structure observed in the collaboration network.

The author “Van der Woulde” (Van DWL) stands out as the most productive and consistent contributor across the entire period (*n* = 59), maintaining a steady stream of publications with notable citation impact, particularly between 2004 (38) and 2022 (39). “Goosey-Tolfrey, Victoria L.” (Goosey-Tolfrey VL) also demonstrates a strong and prolonged research trajectory (*n* = 44), spanning from 2004 (40) and 2024 (41), with multiple years showing elevated citation counts.

Other prominent contributors include “de Groot, Sonja” (De GS) (*n* = 38), “Post, Marcel W.M.” (Post M) (*n* = 24), and “Nash, Mark S.” (Nash M) (*n* = 22), each exhibiting sustained engagement during distinct phases of the study period. De GS shows consistent output across multiple years (42, 43), Post M displays citation peaks between 2008 (44) and 2016 (45) and Nash M's contributions extend from 2004 (46) to 2014 (47, 48), with several highly cited contributions.

Additional contributors such as “Taylor, J. Andrew” (Taylor J) (*n* = 18), “Perret, Catherine” (Perret C) (*n* = 17), “Hunt, Kenneth” (Hunt K) (*n* = 16), and “Nightingale, Thomas” (Nightingale T) (*n* = 16) also show notable publication output, though with comparatively lower citation intensity. Their contributions are often associated with more specialized or network-based collaborations, particularly in areas such as functional rehabilitation and vascular health. “Valent, Linda” (Valent L), despite a lower overall volume (*n* = 16), demonstrates a steady research presence during selected periods, with localized impact and modest citation levels. Notably, several authors maintain active publication records through 2024, indicating continued engagement and sustained contribution to research on rehabilitation and physical exercise in individuals with spinal cord injury.

The map illustrates the scientific collaboration network among authors, constructed based on co-authorship of publications. The network is characterized by low overall density and high modularity, indicating a structure composed of well-defined collaborative communities with limited inter-cluster connectivity. Each node represents an author, and its size reflects the number of publications. Edge thickness indicates the strength of co-authorship ties (number of shared publications), while node proximity reflects collaboration frequency. Cluster colors correspond to modularity-based community detection, grouping authors with strong internal co-authorship intensity.

The co-authorship network comprised 35 authors distributed across six clusters, reflecting the thematic and geographical organization of research on physical exercise and spinal cord injury. Quantitative analysis identified a sparse global structure in which a small subset of authors concentrates structural influence, while most occupy peripheral positions. Van DWL emerged as the most central author, exhibiting the highest PageRank (0.067) and betweenness centrality (197.49), indicating a pronounced bridging role between otherwise weakly connected clusters. Other authors with notable structural relevance included De GS (PageRank = 0.048; betweenness = 79.05), Nash M (betweenness = 40.09), and Bilzon J (betweenness = 23.83), reflecting their role in facilitating information flow across collaborative communities rather than productivity alone. Closeness centrality values were highest for Takken T and De G J (1.0), indicating broad network accessibility despite lower external link strength (Supplementary Table S2).

A highly connected central core is observed around the “Van DWL & Post” axis, which concentrates high productivity, strong internal collaboration, and elevated structural influence, supported by high PageRank and betweenness values. Surrounding this core are consolidated communities such as the “Goosey-Tolfrey” cluster, which forms a cohesive subnetwork with high internal link strength and focuses on applied sports performance (49–52), exercise physiology (53–56), and wheelchair-based training interventions (57–60). This cluster represents a well-established network in adapted sports and exercise physiology, with strong institutional ties to the United Kingdom and Oceania.

As well as the “Nash & Kressler” axis, is mainly based in the United States and emphasizes cardiopulmonary rehabilitation (61–63) and exercise in SCI populations (64, 65), This cluster exhibits moderate-to-high betweenness centrality, reflecting its integrative role between clinical rehabilitation and physiological research streams, and has made important contributions to clinical rehabilitation (66, 67) and physiological responses to training (61, 64, 68). Other clusters exhibit moderate integration and complementary thematic lines, such as the “Nightingale & West” axis, focuses on body composition and vascular health (69–71), representing an emerging interface between clinical research and public health. Peripheral communities, including authors such as Taylor, Picard, Kim, Gater, Gorgey, Takken, De Groot, and Brurrok, display low PageRank and link strength values, indicating specialized niches with limited integration but potential for future incorporation into the broader collaboration network. Overall, the network is characterized by a hub-centered structure with emerging groups and persistent modularity.

Table 3 highlights the concentration of scientific production in high-income countries, with the United States leading, driven primarily by the University of Miami (89 publications), followed by the University of British Columbia in Canada. Institutional collaboration patterns mirror the author-level network, with a small number of institutions concentrating high centrality and link strength. The Netherlands also shows strong representation, with three institutions among the most productive, consistent with prior bibliometric evidence linking sustained investment in clinical and technological research to increased visibility in neurorehabilitation and assistive technologies (72), This distribution reflects the historical predominance of the Europe–North America axis, supported by consolidated infrastructure, stable funding, and specific clinical demands (9, 73, 74). The geographic disparities observed here align with earlier studies (8, 9), and underscore the relevance of expanding international collaboration to integrate underrepresented regions, including Latin America, Asia, and Africa.

**Table 3:** Ranking of the Top 10 universities, their respective countries, and the total publication output from 2004 to 2024 on physical exercise and spinal cord injury.

Affiliation	Articles	Country	Betweenness	Closeness	PageRank
University of Miami	89	United States	25.25	0.0204	0.02818
University of British Columbia	71	Canadá	41.33	0.0250	0.02581
University of Groningen	57	Netherlands	18.95	0.0238	0.02206
University Medical Center Utrecht	33	Netherlands	2.38	0.0182	0.01146
Vrije Universiteit Amsterdan	33	Netherlands	11.13	0.0222	0.01164
University of Bath	26	United Kingdom	15.23	0.0181	0.01056
Havard Medical School	25	United States	17.55	0.0170	0.01011
Loughborough University	25	United Kingdom	21.74	0.0172	0.00988
University of Alabama at Birmingham	24	United States	10.88	0.0164	0.00922

Institutional network analysis identified 38 affiliations, of which ten exhibited the highest PageRank, betweenness, and closeness values, indicating central positions and strong collaborative capacity. The University of Alabama at Birmingham emerged as the most influential institution (PageRank = 0.028), while the University of Miami showed both high PageRank (0.026) and the highest betweenness centrality (41.33), functioning as a principal institutional bridge. Other prominent institutions included the University of Bath (PageRank = 0.022), Leiden University (betweenness = 30.17), and the University of Kansas (betweenness = 15.23). Maastricht University, the University of British Columbia, Ohio State University, and Liverpool John Moores University displayed intermediate yet consistent values, indicating stable integration within specific thematic sub-networks rather than peripheral positioning.

Figure 9 presents the international collaboration network among countries conducting research on PE and SCI. The nodes represent countries, with node size proportional to total publication output, while the edges indicate the intensity of co-authorship links (edge thickness proportional to the number of internationally co-authored publications between countries); spatial proximity reflects the frequency of collaborative interactions. The color-coded clusters highlight regional collaboration patterns and correspond to modularity-based community detection (i.e., collaborative communities identified by stronger within-group than between-group connectivity), helping identify the main scientific production centers and the most active international networks in the field (13, 75).

The analysis identified 23 countries involved in SCI–exercise research, with a strong predominance of high-income nations. The United States constitutes the primary global hub, exhibiting the highest total link strength (TLS = 69). Canada (TLS = 49), England (TLS = 43), the Netherlands (TLS = 41), and the United Kingdom (TLS = 33) form a secondar*y* axis of collaboration, complemented by European countries such as Switzerland, France, and Sweden, which show moderate interaction strength. Brazil (TLS = 15), Saudi Arabia (TLS = 13), and Indonesia (TLS = 7) emerge as developing contributors, positioned in the network periphery and linked predominantly through collaborations with central hubs in North America and Western Europe, rather than through dense South–South connections. This pattern indicates a geographically concentrated collaboration structure, consistent with a low-density, high-modularity international network.

The United States, represented by the largest node in the network, stands out for its high connectivity, as reflected by its publication volume and elevated international link strength, which support its role as a central hub (8–10). Although contextual factors such as research infrastructure and technological development may contribute to this positioning, the present results are interpreted primarily through the network metrics (node size and link strength) rather than external explanatory variables. Previous studies have similarly identified the United States as the global leader in the field, followed by Canada, Australia, the Netherlands, and Switzerland (8). Canada occupies a strategic position by linking North America and Europe, whereas the Netherlands maintains considerable influence due to specialized rehabilitation centers and high scientific productivity (7–10).

The contrast between the dense central core and the peripheral positioning of low- and middle-income countries indicates asymmetries in the distribution and connectivity of scientific production. Liu et al. (9), have previously noted the dominance of the Global North and the limited participation of regions such as Latin America, Africa, and parts of Asia, which constrains the applicability of research findings to diverse socioeconomic contexts. Phadke et al. (10) reinforce this pattern by showing that technological advances in rehabilitation, such as robotic therapies, remain concentrated in core countries, in network terms, these disparities are expressed by lower total link strength and reduced connectivity for underrepresented regions, indicating limited integration into high-throughput international collaboration pathways. Although the current collaboration landscape is highly productive, expanding integration with underrepresented regions may increase network density and reduce modular fragmentation, strengthening global research networks and improving the representativeness and transferability of evidence for individuals with SCI across diverse settings.

### 3.4 Research trends

Figure 6 displays the distribution of trending topics in scientific literature on physical exercise (PE) and spinal cord injury (SCI) from 2004 to 2024. Each line represents a keyword, while the circles indicate the period in which that term appeared most frequently in the literature. Circle size is proportional to term frequency, allowing direct visualization of the relative prominence of each keyword over time (Supplementary Table S5).

The period from 2004 to 2012 was marked by the consolidation of physiological and methodological foundations. During this phase, the most frequent terms included *paraplegia* (*n* = 141), *muscle contraction* (*n* = 30), *exercise tolerance* (*n* = 29), *human experiment* (*n* = 29), *kinematics* (*n* = 6), and *ergometer* (*n* = 20), indicating a strong emphasis on physiological testing, instrumentation, and exploratory clinical experimentation. This phase is associated with early methodological and physiological research patterns (76–79). The presence of terms such as *kinematics, ergometer*, and *functional assessment* highlights a strong focus on instrumentation, biomechanical analysis, and the evaluation of physical capacity in individuals with SCI (78, 80–83). Additionally, terms such as *paraplegics*, *human experiment*, and *case report* suggest the predominance of exploratory clinical studies, typical of an early-stage scientific field (84–86).

The 2013–2017 period reflects thematic expansion and the beginning of clinical consolidation. Frequently used keywords included *spinal cord injury* (*n* = 243), *exercise* (*n* = 323), *fitness* (*n* = 116), *performance* (*n* = 76), *aerobic capacity* (*n* = 44), *reliability* (*n* = 38), and *procedures* (*n* = 41). These terms indicate a clear shift toward more clinically applied topics, with PE gaining prominence as both a therapeutic and preventive strategy for individuals with SCI (87–93). Compared with the previous period, this stage shows higher keyword recurrence and stronger co-occurrence density, indicating increased keyword recurrence and co-occurrence density. The rise of terms like *aerobic capacity, performance*, and *fitness* indicates increased interest in structured physical training responses, while *pathophysiology* and *reliability* reflect methodological refinement and growing scientific rigor (94–97).

In the 2018–2020 period, the literature shows a transition toward public health and functionality, with frequently used terms including adults (*n* = 57), people (*n* = 54), *cardiorespiratory fitness* (*n* = 44), *quality of life, six-minute walk test, cohort analysis, wheelchair users,* and *resistance training* (*n* = 22). The increased frequency and co-occurrence strength of population-level and functional outcome terms suggest a shift toward longitudinal, epidemiological, and clinically meaningful endpoints (98–103). At this stage, clusters related to quality of life (102, 104) and resistance training began to gain prominence, indicating the emergence of broader health-oriented perspectives within the field (98, 105).

Finally, the 2021–2024 period is characterized by clinical specialization and a cardiometabolic focus, with predominant terms such as *low-density lipoprotein cholesterol* (*n* = 7), *high-density lipoprotein cholesterol* (*n* = 7), *systolic blood pressure* (*n* = 12), *diastolic blood pressure* (*n* = 10), *questionnaire* (*n* = 15), and the *American Spinal Injury Association Impairment Scale* (ASIA) (77, 106–108). These findings indicate a trend toward greater clinical sophistication and specialization. Keywords related to cardiovascular and metabolic health (e.g., *LDL, HDL, systolic blood pressure*) reflect alignment with current public health concerns, recognizing the elevated risk of chronic diseases in people with disabilities. The use of standardized instruments such as the *ASIA Impairment Scale* and validated questionnaires (109–113) illustrates the consolidation of functional assessment protocols for the SCI population. This trajectory reflects not only the scientific maturation of the field but also the responsiveness to the real needs of a population with high morbidity and mortality risks associated with physical inactivity and chronic functional limitations (114–117). Although these terms represent a relatively small proportion of total keywords (each accounting for <1.5% of all keyword occurrences), their recent temporal concentration and increasing co-occurrence strength indicate emerging and specialized research fronts rather than marginal themes. The use of standardized instruments and cardiometabolic markers reflects increasing clinical sophistication and alignment with chronic disease risk management in SCI populations.

The keyword co-occurrence network (Figure 10) identified five thematic clusters composed of terms that frequently appear together in the literature (13, 75). Each node represents a keyword, with its size indicating the frequency and thematic relevance of that term. The proximity between nodes reflects the degree of similarity based on co-occurrence, calculated using the VOS algorithm. The edges (links) connecting terms are thicker when co-occurrence frequency is higher.

The clusters, differentiated by color, group together terms that are closely related, revealing thematic subfields and potential interdisciplinary relationships. This visualization enables the identification of consolidated research areas, emerging trends, and possible gaps within the field. The conceptual mapping thus provides insight into how the scientific discourse is structured and how key concepts are interlinked across publications.

The red cluster representing 159 terms (35.5% of all clustered keywords), is centered on the terms “*Exercise*,” “*Fitness*,” “*Performance*,” “*Health*,” “*Individuals*,” “*Cardiorespiratory Fitness*,” and “*Spinal Cord Injury*”, and can be characterized as the “Physical Performance and Functional Capacity in Individuals with SCI” axis. These keywords reflect research focused on exercise programs aimed at improving cardiorespiratory fitness (118), general health outcomes (93), and physical performance in wheelchair users (76), as demonstrated in foundational studies such as Jacobs & Nash (46).

The green cluster, includes 90 terms (19.9%), represented by terms such as “*Exercise Tolerance*,” “*Lung Ventilation*,” “*Respiration*,” “*Posture*,” “*Lactic Acid*,” “*Pulmonary Ventilation*,” and “*Human Experiment*”, corresponds to the “Physiological Assessment and Respiratory Responses to Exercise” axis. This research line encompasses studies evaluating exercise tolerance (119), pulmonary ventilation (120), and metabolic responses under controlled experimental settings (121).

The blue cluster, with 76 terms (16.8%), focuses on like “*Clinical Article*,” “*Treatment Outcome*,” “*Middle Aged*,” “*Hospital Discharge*,” “*Walking Speed*,” “*Electrotherapy*,” and “*Kinesiotherapy*”, is associated with the “Clinical Rehabilitation and Therapeutic Approaches” axis. This group comprises clinical trials (122), functional physiotherapy interventions (88, 123), and rehabilitation strategies for individuals with SCI (124).

The yellow cluster (63 terms; 14.0%) features terms such as “*Wheelchair*,” “*Wheelchairs*,” “*Biomechanics*,” “*Human Engineering*,” “*Equipment Design*,” “*Propulsion*,” and “*Instrumentation*”, and can be classified under the “Applied Engineering and Wheelchair Biomechanics” axis. Studies within this domain typically address ergonomics (125), propulsion kinematics (126), and assistive device design (39, 127). Finally, the purple cluster (63 terms;14.0%) encompasses terms including “*Body Composition*,” “*Resistance Training*,” “*Metabolism*,” “*Obesity*,” “*Health Promotion*,” “*Motivation*,” “*Insulin Sensitivity*,” and “*Nutrition*”, representing the “Body Composition, Metabolism, and Health Promotion” axis. Publications within this cluster explore the effects of physical exercise on body composition, metabolic profile, and quality of life in individuals with SCI (24, 69, 110).

While the axes related to physiological assessment, physical performance, and functional rehabilitation are extensively developed, areas such as assistive engineering applied to sports and the metabolic integration of exercise prescription for wheelchair users remain underexplored. Quantitatively, keywords associated with psychosocial and behavioral aspects (e.g., “motivation,” “mental health,” and “self-efficacy”) appeared in fewer than 10 documents each, representing less than 1.5% of all author keywords and occupying peripheral positions in the co-occurrence network (average link strength <2). Similarly, terms linked to assistive technology (“virtual reality,” “exoskeleton,” “robotics”) accounted for approximately 2% of all keyword occurrences, contrasting with over 30% concentration in clusters related to physiological and performance outcomes. This imbalance indicates that technological and psychosocial domains occupy peripheral positions in the co-occurrence network.

The MCA analysis identified 43 high-frequency conceptual terms distributed across four conceptual regions, each defined by their position along the first two factorial dimensions and by their proportional representation in the conceptual space (Supplementary Table S9). These regions comprised: a technical-clinical region (12 terms; 27.9%), associated with therapeutic interventions, biomechanics, and functional assessment; a health/performance region (14 terms; 32.5%), related to physiological responses and the effects of physical activity on the health of people with disabilities; a central integrative region (10 terms; 23.2%), linking rehabilitation, aerobic capacity, and exercise physiology; and an upper physiological region (7 terms; 16.2%), composed of terms referring to population characteristics and cardiorespiratory variables. Together, these proportions indicate that the conceptual structure of the field is predominantly organized around clinical intervention, functional performance, and physiological response axes, whereas population descriptors and test-specific variables occupy more peripheral yet complementary positions.

The two principal conceptual axes further reinforce this structure. The first dimension (Dim 1) reflects a technical–clinical gradient, grouping terms related to therapeutic procedures, functional assessment, controlled studies, and kinesiotherapy, indicating strong contributions from experimental and clinically oriented rehabilitation research. The second dimension (Dim 2) captures a health–performance gradient, encompassing concepts associated with physical activity, cardiorespiratory fitness, performance, and adaptive outcomes in individuals with spinal cord injury. The orthogonal configuration of these dimensions supports a multidimensional conceptual organization rather than a unidirectional thematic hierarchy (Supplementary Table S8).Within the central integrative region, high-frequency and centrally positioned terms such as exercise, aerobic capacity, physical fitness, training, metabolism, and physiology exhibit minimal distance from the origin of the factorial space, acting as convergence points between rehabilitation science, exercise physiology, and exercise-based interventions for populations with reduced mobility (20, 58, 65, 81, 100). This central positioning reflects strong co-occurrence and conceptual connectivity rather than thematic dominance, consistent with their central positioning and high co-occurrence across multiple thematic regions. In contrast, the upper physiological/population-specific region, defined by terms such as young adult, adolescent, heart rate, oxygen consumption, paraplegia, and wheelchair, occupies a more peripheral position in the MCA map, indicating lower relative frequency and weaker connectivity with the core conceptual axis (Supplementary Table S9). These terms represent specialized subdomains focused on physiological specificity and population stratification, complementing, rather than driving, the central conceptual structure of the field (128).

Overall, the mapping demonstrates that the field is organized around multidimensional approaches combining functional assessment (71, 106, 110), cardiorespiratory rehabilitation (95, 118), PE-based interventions and their effects on health and performance (28, 119), particularly among wheelchair users. These findings align with recent work on training in adapted sports (129), cardiovascular adaptations to exercise in individuals with SCI (46), and the impact of physical activity on cardiorespiratory capacity and quality of life. However, areas such as standardization of therapeutic protocols, kinesiotherapy, and specific rehabilitation procedures remain underdeveloped, as do psychosocial themes and assistive technology, which comprise less than 5% of co-occurring terms. Advancing the field will require deeper exploration of: (i) functional assessment and cardiorespiratory responses to exercise, (ii) the effects of physical activity on the health and performance of wheelchair users, and (iii) the development of robust rehabilitation protocols that simultaneously integrate psychosocial, technological, and contextual factors still marginalized in the literature.

## 4 Limitations

This study presents several methodological limitations that should be considered when interpreting its findings. First, scientometric analyses are inherently dependent on indexed and published material; therefore, the results reflect database coverage rather than the entirety of scientific knowledge produced in the field. Differences in indexing policies, update frequency, and journal inclusion across PubMed, Scopus, and Web of Science may introduce coverage bias, favoring regions, journals, or research groups with greater visibility in these platforms. Second, although a standardized search string and harmonized field tags were applied across databases, the keyword-driven nature of bibliographic searches may lead to the omission of studies that use alternative terminology, incomplete metadata, or non-standardized descriptors. Variations in indexing quality and metadata structure remain intrinsic limitations of scientometric data extraction.

Third, the selected time frame (2004–2024) restricts conclusions to this period and does not encompass earlier foundational literature or the most recent publications still pending indexing. This is particularly relevant for the year 2024, for which publication counts and citation indicators are likely underestimated due to indexing delays, constituting a time-lag bias commonly observed in bibliometric studies. Accordingly, results referring to the most recent year should be interpreted cautiously, as database updates may not yet fully reflect the complete volume of publications. Although studies were not restricted by language during screening, the reliance on large international databases may still underrepresent scientific output from regions whose journals are not systematically indexed in PubMed, Scopus, or Web of Science.

Fourth, this analysis intentionally excluded dissertations, theses, conference proceedings, reports, and other forms of grey literature. While this decision enhances methodological consistency and comparability of citation-based metrics by focusing on peer-reviewed journal articles, it may result in the omission of emerging, locally disseminated, or practice-oriented research not yet represented in indexed journals. Fifth, despite calibration procedures and duplicate screening conducted by two independent reviewers, the possibility of misclassification or inconsistencies during automated and manual filtering cannot be entirely excluded, particularly when differentiating structured exercise interventions from broader rehabilitation protocols.

Finally, the interpretation of collaboration networks, co-citation structures, and thematic clusters is exploratory and descriptive. These visualizations capture relational patterns within metadata but do not allow causal inference or assessment of methodological rigor, quality, or clinical impact of individual studies. Consequently, all conceptual and structural interpretations should be understood within the inherent boundaries of scientometric methodology.

## 5 Conclusion

This scientometric analysis provides a comprehensive mapping of two decades of global research on physical exercise and spinal cord injury, based on quantitative indicators of productivity, collaboration, and thematic structure. The results demonstrate that the field is predominantly organized around physiological, functional, and clinical rehabilitation domains, which together account for the majority of high-frequency keywords and form the most mature and densely connected thematic clusters. In contrast, psychosocial dimensions, behavioral determinants, and assistive-technology–related topics occupy peripheral positions in the co-occurrence and conceptual networks, representing a small proportion of total keywords and exhibiting lower co-occurrence strength. These patterns indicate not an absence of such themes, but rather their limited integration into the dominant research structure observed over the last two decades.

The primary contribution of this study lies in systematically identifying the structural composition of the field, the relative maturity of its thematic domains, and the persistence of underrepresented research fronts through an integrated, multi-database scientometric approach. By combining temporal trend analysis, collaboration networks, keyword co-occurrence, and multiple correspondence analysis, this mapping clarifies how research priorities have evolved and where structural gaps remain. Future research directions should be grounded in these observed gaps, particularly by promoting studies that integrate psychosocial outcomes, behavioral factors, and assistive technologies with established physiological and functional frameworks. Additionally, the marked concentration of scientific production and collaboration in high-income countries highlights the need for broader international participation, especially from low- and middle-income regions, to enhance the diversity and applicability of evidence. Continued scientometric monitoring may help track whether these underrepresented domains become more structurally integrated as the field evolves.

## Data Availability

The raw data supporting the conclusions of this article will be made available by the authors, without undue reservation.
