# Supplementary material for: Global trends in scientific production on physical exercise and spinal cord injury
Source: Front Sports Act Living. 2026 Jan 22;7:1678162. doi: 10.3389/fspor.2025.1678162 (PMC12872875; doi:10.3389/fspor.2025.1678162)
Supplement: Supplementary file 1 [file Datasheet1.pdf]

Supplementary Material S1 – Complete Search Strategy

Web of Science (TS = Topic)

Search string:

TS = ("wheelchair" OR "wheelchair users" OR "wheelchair-bound" OR "wheelchair dependent" OR "wheelchair athletes" OR "paralympic athletes" OR "adapted sports" OR "wheelchair sports" OR paraplegia OR tetraplegia OR quadriplegia OR "spinal cord injury" OR "lower extremity paralysis" OR "physical disabilit*" OR "people with disabilities" OR "persons with disability" OR "mobility impairment" OR "neurological impairment") AND ("exercise" OR "aerobic exercise" OR "physical exertion" OR "exercise tolerance" OR "cycle ergometer" OR "arm ergometer" OR "arm crank ergometer" OR "upper-body exercise" OR "wheelchair propulsion test" OR ergonom*) AND ("physical fitness" OR "aerobic capacity" OR "aerobic power" OR "cardiorespiratory fitness" OR "cardiopulmonary exercise test" OR "submaximal test" OR "oxygen consumption" OR "VO2 max" OR "VO2max" OR "VO2peak" OR "maximal aerobic capacity" OR "cardiorespiratory assessment")

Indexes searched: SCI-EXPANDED, SSCI, ESCI
Timespan: 2004–2024
Language: No restrictions
Date of search: 10 June 2025
Export format: BIB

PubMed (Title/Abstract Search)

Search string:

("wheelchair" OR "wheelchair users" OR "wheelchair-bound" OR "wheelchair dependent"

OR "wheelchair athletes" OR "paralympic athletes" OR "adapted sports" OR "wheelchair sports" OR paraplegia OR tetraplegia OR quadriplegia OR "spinal cord injury" OR "lower extremity paralysis" OR "physical disabilit*"OR "people with disabilities" OR "persons with disability" OR "mobility impairment" OR "neurological impairment") AND ("exercise" OR "aerobic exercise" OR "physical exertion" OR "exercise tolerance" OR "cycle ergometer" OR "arm ergometer" OR "arm crank ergometer" OR "upper-body exercise" OR "wheelchair propulsion test" OR ergonom*) AND ("physical fitness" OR "aerobic capacity" OR "aerobic power" OR "cardiorespiratory fitness" OR "cardiopulmonary exercise test" OR "submaximal test" OR "oxygen consumption" OR "VO2 max" OR "VO2max" OR "VO2peak" OR "maximal aerobic capacity" OR "cardiorespiratory assessment")

Fields searched: Title/Abstract
Filters applied: Publication years 2004–2024
Language: No restrictions
Date of search: 10 June 2025
Export format: TXT

Scopus (TITLE-ABS-KEY)

Search string:

TITLE-ABS-KEY (("wheelchair" OR "wheelchair users" OR "wheelchair-bound" OR "wheelchair dependent" OR "wheelchair athletes" OR "paralympic athletes" OR "adapted sports" OR "wheelchair sports" OR paraplegia OR tetraplegia OR quadriplegia OR "spinal cord injury" OR "lower extremity paralysis" OR "physical disabilit*" OR "people with disabilities" OR "persons with disability" OR "mobility impairment" OR "neurological impairment") AND ("exercise" OR "aerobic exercise" OR "physical exertion" OR "exercise tolerance" OR "cycle ergometer" OR "arm ergometer" OR "arm crank ergometer" OR "upper-body exercise" OR "wheelchair propulsion test" OR ergonom*) AND ("physical fitness" OR "aerobic capacity" OR "aerobic power" OR "cardiorespiratory fitness" OR "cardiopulmonary exercise test" OR "submaximal test" OR "oxygen consumption" OR "VO2 max" OR "VO2max" OR "VO2peak" OR "maximal aerobic capacity" OR "cardiorespiratory assessment"))

Filters applied: Publication years 2004–2024
Language: No restrictions
Date of search: 10 June 2025
Export format: BIB

Table S2 - Top 10 most influential authors on the web (according to PageRank)

Authors	Cluster	Betweenness	Closeness	PageRank
Van DWL	5	197.49	0.0159	0.0672
De G S	5	79.05	0.0154	0.0481
Post M	5	3.94	0.0116	0.0385
Valent L	5	14.70	0.0130	0.0291
Bilzon J	6	23.83	0.0101	0.0245
Dallmeijer A	5	1.66	0.0115	0.0217
Nash M	6	40.09	0.0125	0.0205
Kouwijzer I	5	14.78	0.0143	0.0204
Takken T	1	0.00	1.0000	0.0200
De GJ	1	0.00	1.0000	0.0200

PageRank is the best metric available in the archive for identifying structural importance; Betweenness and Closeness were also included.)

AFFILIATIONS

Table S3. Top 10 most influential institutions (PageRank)

Affiliations	Betweenness	Closeness	PageRank
**Univ Alabama Birmingham**	**25.25**	0.0204	**0.02818**
**Univ Miami**	41.33	0.0250	0.02581
**Univ Bath**	18.95	0.0238	0.02206
**Aalborg Univ**	2.38	0.0182	0.01146
**Liverpool John Moores Univ**	11.13	0.0222	0.01164
**Univ Leiden**	30.17	0.0189	0.01124
**Univ Kansas**	15.23	0.0181	0.01056

Affiliations	Betweenness	Closeness	PageRank
**Maastricht Univ**	17.55	0.0170	0.01011
**Univ British Columbia**	21.74	0.0172	0.00988
**Ohio State Univ**	10.88	0.0164	0.00922

Countries

Table S4. Top Countries (Top 10) According To Total Link Strength (TLS)

Rank	Country	Documents	Citations	Total Link Strength (TLS)
1	USA	210	67	69
2	Canada	74	1571	49
3	England	49	137	43
4	Netherlands	102	80	41
5	United Kingdom	63	51	33
6	Switzerland	29	229	24
7	Australia	24	410	21
8	Brazil	34	297	15
9	Saudi Arabia	6	104	13
10	France	22	894	11
11	Poland	17	460	10
12	Malaysia	11	41	9
13	Italy	23	40	8
14	South Korea	22	282	8
15	Denmark	11	138	8
16	Indonesia	6	278	7
17	Japan	32	58	6
18	Ireland	7	152	5

Rank	Country	Documents	Citations	Total Link Strength (TLS)
19	Sweden	8	42	3
20	Germany	11	366	3
21	Norway	18	29	3
22	Belgium	7	178	1
23	India	6	62	0
24	Turkey	7	160	0

These ten countries form the core of global collaboration in this area, accounting for most of the international connections in the field. (TLS = best metric for international collaboration, according to VOSviewer standard)

Supplementary Material - Research Trends

Trending Topics in Scientific Production on Physical Exercise and Spinal Cord Injury

Table S5. Trending Topics in Scientific Production on Physical Exercise and Spinal Cord Injury by frequency and year

item	freq	year_q1	year_med	year_q3
calorimetry	7	2005	2005	2019
kinematics	6	2005	2005	2011
acceleration	5	2005	2005	2019
statistical significance	10	2004	2006	2006
physical stress	7	2004	2006	2009
biomedical engineering	6	2006	2006	2008
ergometer	20	2006	2007	2011
paraplegics	9	2004	2007	2013
behavior	5	2005	2007	2017
muscle contraction	30	2006	2008	2013
analysis of variance	20	2004	2008	2010
adaptation	17	2007	2008	2013
exercise tolerance	29	2005	2009	2019
human experiment	29	2006	2009	2020
gait	25	2005	2009	2017
priority journal	79	2007	2010	2015
skeletal	32	2007	2010	2016
endurance	31	2006	2010	2020
paraplegia	141	2007	2011	2018
exercise therapy	64	2008	2011	2016
physical fitness	57	2009	2011	2013
adult	426	2008	2012	2018
humans	272	2009	2012	2018
article	254	2008	2012	2018
male	450	2009	2013	2018
oxygen consumption	344	2008	2013	2018
human	280	2008	2013	2018
female	297	2009	2014	2018
heart rate	190	2008	2014	2019
spinal cord injuries	162	2009	2014	2019
exercise	323	2010	2015	2020
spinal cord injury	243	2009	2015	2020
middle aged	197	2010	2015	2019
performance	70	2011	2016	2021
capacity	44	2013	2016	2020
responses	42	2012	2016	2019
spinal-cord-injury	64	2012	2017	2020
physical activity	51	2011	2017	2021
reliability	38	2012	2017	2020
people	54	2013	2018	2021
physical-activity	49	2014	2018	2021

procedures	41	2016	2018	2021
adults	57	2017	2019	2022
blood pressure	38	2014	2019	2022
exercise intensity	33	2011	2019	2022
cardiorespiratory fitness	44	2016	2020	2023
health	33	2017	2020	2023
validity	18	2017	2020	2021
strength	31	2015	2021	2022
muscle fatigue	21	2010	2021	2022
insulin	15	2019	2021	2023
resistance training	22	2018	2022	2023
questionnaire	15	2014	2022	2023
systolic blood pressure	12	2017	2022	2024
diastolic blood pressure	10	2013	2023	2024
high density lipoprotein cholesterol	7	2015	2023	2024
low density lipoprotein cholesterol	7	2016	2023	2024

Co-Occurrence Network of Author Keywords on Physical Exercise and Spinal Cord Injury

Table S6. Distribution of words, terms, and thematic synthesis. Co-occurrence network of the author's keywords on physical exercise and spinal cord injury

Cluster (Color)	Number of Terms	% of the Analysis (5 Clusters)	Main Terms	Thematic Synthesis
Red	159	35.3%	*Exercise, Fitness, Performance, Health, Individuals, Cardiorespiratory Fitness, Spinal Cord Injury*	"Cardiorespiratory Fitness," and "Spinal Cord Injury"
Green	90	19.9%	*Exercise Tolerance, Lung Ventilation, Respiration, Posture, Lactic Acid, Pulmonary Ventilation, Human Experiment*	"Physiological Assessment and Respiratory Responses to Exercise"
Blue	76	16.8%	*Clinical Article, Treatment Outcome, Middle Aged, Hospital Discharge, Walking Speed, Electrotherapy, Kinesiotherapy*	"Clinical Rehabilitation and Therapeutic Approaches"
Yellow	63	14.0%	*Wheelchair, Wheelchairs, Biomechanics, Human Engineering, Equipment Design, Propulsion, Instrumentation*	"Applied Engineering and Wheelchair Biomechanics"
Purple	63	14.0%	*Body Composition, Resistance Training, Metabolism, Obesity, Health Promotion, Motivation, Insulin Sensitivity, Nutrition*	"Body Composition, Metabolism, and Health Promotion"

Percentages calculated based solely on the five interpreted clusters (total = 451 terms)

Table S7. Frequency of occurrence of the main thematic terms identified in the co-occurrence analysis.

Terms	***Ocorrence***
Exercise	**345**
Fitness	**116**
Performance	**76**

Health	**43**
Individuals	**115**
Cardiorespiratory Fitness	**58**
Spinal Cord Injury	**281**
Exercise Tolerance	**24**
Lung Ventilation	**17**
Respiration	**9**
Posture	**7**
Lactic Acid	**27**
Pulmonary Ventilation	**14**
Human Experiment	**29**
Clinical Article	**124**
Treatment Outcome	**31**
Middle Aged	**115**
Hospital Discharge	**6**
Walking Speed	**12**
Electrotherapy	**10**
Kinesiotherapy	**42**
Wheelchair	**108**
Wheelchairs	**92**
Biomechanics	**36**
Human Engineering	**5**
Equipment Design	**12**
Propulsion	**23**
Instrumentation	**6**
Body Composition	**27**
Resistance Training	**22**
Metabolism	**52**
Obesity	**21**
Health Promotion	**13**

Motivation	**5**
Insulin Sensitivity	**11**
Nutrition	**6**

Table S8. Conceptual Structure Map from Multiple Correspondence Analysis (MCA).

Word	Dim.1	Dim.2	Cluster
male	1	-0,2	1
adult	1,03	-0,13	1
oxygen.consumption	1,05	-0,19	1
exercise	0,11	0,02	1
female	1,08	-0,08	1
human	1,03	-0,05	1
humans	0,95	-0,18	1
article	1,05	0,06	1
spinal.cord.injury	1,15	0,45	1
middle.aged	1,17	0,24	1
exercise.test	1,11	-0,28	1
heart.rate	1,17	-0,25	1
spinal.cord.injuries	1,28	0,54	1
paraplegia	0,76	0,24	1
clinical.article	1,23	0,64	1
controlled.study	1,15	0,19	1
individuals	-1	0,27	1
fitness	0,02	0	1
physiology	1,12	-0,15	1
young.adult	1,11	-1,04	1
quadriplegia	1,06	0,47	1
wheelchairs	0,86	-1,85	1
wheelchair	0,76	-1,64	1
priority.journal	1,23	0,47	1
pathophysiology	1,37	0,54	1
performance	-0,86	0,1	1
muscle.strength	0,92	0,54	1
adolescent	1,14	-0,65	1
muscle	0,55	0,5	1
exercise.therapy	1,33	1,23	1
spinal.cord.injury.1	-0,99	0,23	1
arm	0,67	-0,44	1
ergometry	0,63	0,19	1
adults	-1	0,32	1
physical.fitness	1,05	-0,14	1
people	-1,03	0,31	1
metabolism	0,77	0,83	1
physical.activity	1,13	0,3	1
physical.activity.1	-1,03	0,3	1
capacity	-1	0,21	1
kinesiotherapy	1,38	1,41	1
responses	-0,98	0,09	1
procedures	1,49	0,64	1

Table S9. Conceptual regions of the MCA map: number of terms, percentages, core terms, and thematic synthesis.

Conceptual Region (MCA)	Number of Terms	% of Total (n = 43)	Top Terms (up to 10)	Thematic Synthesis
1. Technical–Clinical Axis (Dim 1 negative / Dim 2 negative)	12	27.9%	*kinesiotherapy, exercise therapy, procedures, pathophysiology, clinical article, controlled study, muscle strength, quadriplegia, ergometry, spinal cord injuries*	Focused on therapeutic interventions, biomechanics, clinical experimentation, and functional assessment.
2. Health–Performance Axis (Dim 1 positive)	14	32.5%	*performance, responses, capacity, physical activity, rehabilitation, spinal cord injury, cardiorespiratory fitness, fitness, health, individuals*	Emphasizes functional capacity, physiological responses, and health-related outcomes in individuals with disabilities.
3. Central Integrative Region (Dim 1 around 0)	10	23.2%	*exercise, aerobic capacity, physical fitness, training, muscle, walking, metabolism, adults, humans, physiology*	Integrates rehabilitation, physiological performance, and exercise-based interventions.
4. Upper Physiological / Population-Specific Region	7	16.2%	*young adult, adolescent, heart rate, oxygen consumption, exercise test, arm, wheelchair, paraplegia*	Reflects physiological responses, population characteristics, and cardiorespiratory variables.
